# CXCR4/SDF1 mediate hypoxia induced chondrosarcoma cell invasion through ERK signaling and increased MMP1 expression

**DOI:** 10.1186/1476-4598-9-17

**Published:** 2010-01-26

**Authors:** Xiaojuan Sun, Lei Wei, Qian Chen, Richard M Terek

**Affiliations:** 1 Department of Orthopaedics, Warren Alpert Medical School of Brown University and Rhode Island Hospital, Providence RI, USA

## Abstract

**Background:**

Chondrosarcoma is a disease that does not respond to conventional cytotoxic chemotherapy and expression of MMP1 is a marker for a poor prognosis. The mechanism of increased MMP1 expression in chondrosarcoma is not completely known. Our goal is to identify molecular pathways that could serve as therapeutic targets. Chondrosarcoma become hypoxic as they grow, are capable of eliciting an angiogenic response, and typically metastasize to the lungs. The present study determined the effect of hypoxia and specifically HIF-1a on expression of CXCR4 and MMP1 and their role in chondrosarcoma cell invasion.

**Results:**

CXCR4 and its ligand, SDF1, are upregulated in primary chondrosarcoma tumors compared to normal articular cartilage, and CXCR4 was upregulated in chondrosarcoma cell line JJ compared to normal chondrocytes. Hypoxia and specifically HIF-1a increased CXCR4 and MMP1 expression in JJ cell line and chondrosarcoma invasion *in vitro*. The hypoxia mediated increase in MMP1 expression and chondrosarcoma invasion could be inhibited by siRNA directed at HIF-1a or CXCR4, the CXCR4 inhibitor AMD3100, as well as with ERK inhibitor U0126 and ERK siRNA.

**Conclusions:**

Chondrosarcoma cell invasion is increased by hypoxia induced expression of CXCR4 and MMP1 and is mediated by HIF-1a and ERK. Both invasion and MMP1 can be inhibited with CXCR4 blockade, suggesting that CXCR4/SDF1 signaling may be a therapeutic target for chondrosarcoma.

## Background

Chondrosarcoma is the second most common primary malignant bone. It is a rare disease with a poor prognosis, usually occurs in adults, and the cure rate for this disease has not improved over the last several decades [1,2]. For high grade tumors the cure rate has remained at 10-25%[3]. The treatment for chondrosarcoma is surgical resection; chemotherapy and radiation therapy are not ordinarily used since chondrosarcoma are resistant to these adjuvant modalities. In contrast to chondrosarcoma, osteosarcoma, which usually occurs in adolescents, is sensitive to chemotherapy and the cure rate has increased from 20% to 75% with the advent of multiagent chemotherapy. However, in patients with either tumor, the majority of those who are not cured succumb to lung metastases. Our efforts are directed at elucidating the mechanisms of chondrosarcoma invasion and metastasis.

Invasion, angiogenesis, migration, and metastasis are intertwined processes regulated by overlapping molecular pathways. Chemokines and their receptors compose one such pathway and are involved with cell trafficking, migration, and proliferation. There are four groups of chemokine receptors: C, CC, CXC, and CX3C. Chemokine receptor four (CXCR4) is a seven-transmembrane G-protein-coupled receptor, whose activation leads to intracellular signaling cascades. CXCR4 is expressed in dendritic cells, naïve T cells, NK cells, and monocytes and is also the chemokine receptor most commonly expressed in tumors. Within normal cells chemokine receptors are important in immune cell function and migration of stem cells to sites of injury. Within tumor cells, chemokine receptor expression is related to development of metastases preferentially to sites with expression of the corresponding chemokine[4]. The ligand for CXCR4 is the chemokine stromal cell derived factor one (SDF1) which is expressed in the lung and other sites of metastases. CXCR4/SDF1 also indirectly promotes tumor metastasis by mediating proliferation and migration of tumor cells and enhancing tumor-associated angiogenesis [5]. The expression of chemokine receptors has been mostly investigated in carcinoma and increased levels of expression have been found in breast, gastric, colorectal, and lung cancer. CXCR4 expression has also been studied in melanoma, chondrosarcoma, and osteosarcoma. In the latter expression of CXCR4 correlates with overall survival, event-free survival, and metastasis free survival [6] For review see [7,8].

Another factor that drives aggressive behavior in cancer is hypoxia. Hypoxia is a signal that develops as tumors outgrow their blood supply and results in a large number of adaptive changes aimed at surviving in the hypoxic environment as well as correcting the oxygen deficit. HIF-1 is a dimeric transcription factor composed of HIF-1 alpha and beta subunits. HIF-1 protein levels increase as a result of decreased degradation of the oxygen sensitive subunit HIF-1alpha. HIF-1 modulates changes in gene expression during hypoxia. One of the better characterized phenotypic changes induced by hypoxia is angiogenesis, largely mediated by HIF-1 and vascular endothelial growth factor (VEGF) which increases vessel ingrowth from surrounding tissue into the tumor. Our prior work has shown that grades II and III chondrosarcoma express higher levels of HIF-1 and VEGF than benign and grade I cartilage tumors[9] Grades II and III chondrosarcoma are the tumors that metastasize and have poor survival. Hypoxia is also known to increase CXCR4 expression in other systems [10].

Tissue invasion by tumor cells and tumor induced blood vessels also requires matrix metalloproteinases. Specific tumors preferentially express different MMPs. In chondrosarcoma, MMP1 is the dominant metalloproteinase that is expressed and is a marker for poor prognosis [11]. However, the mechanisms of increased MMP1 expression in chondrosarcoma are incompletely understood.

Therefore, we investigated the expression of CXCR4 in normal chondrocytes, normal cartilage, chondrosarcoma tissue, and chondrosarcoma cells (CS) and hypothesized that CXCR4 is overexpressed in chondrosarcoma, is upregulated by hypoxia and specifically by HIF-1, and increases the invasive phenotype by increasing expression of MMP1.

## Results

### SDF1 and CXCR4 expression are increased in primary chondrosarcoma

**Figure 1:**
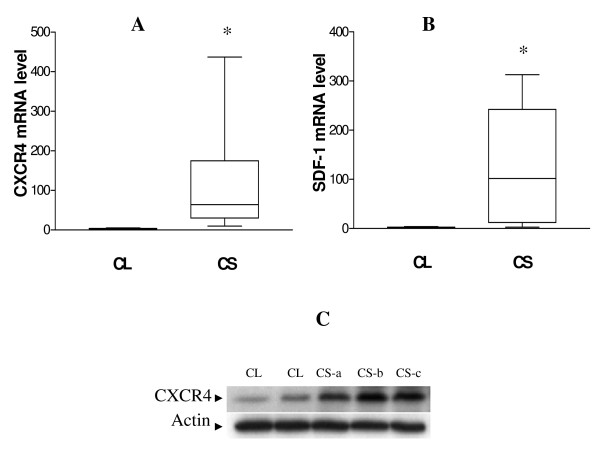
**Expression of CXCR4 and SDF-1 in normal cartilage (CL) and chondrosarcoma tissue (CS)**. A and B, the endogenous mRNA levels of CXCR4 and SDF-1were quantified by qRT-PCR and normalized to 18S. Graphs represent the median of CXCR4 and SDF-1 levels with the 25th-75th percentiles, *, p < 0.001, CS (n = 16) versus CL (control, n = 8). C, CXCR4 protein level in CL and CS. Whole cell lysates from two CL and three CS specimens were subject to Western blot to detect CXCR4 protein. Representative blot is shown from one of three independent experiments. Actin was used as the loading control.

As a first step in evaluating the potential role of SDF1 and CXCR4 in chondrosarcoma biology, we analyzed primary chondrosarcoma tissue and articular cartilage for expression of mRNA and protein for these genes using qRT-PCR and Western blotting. We found that the median CXCR4 and SDF1 mRNA levels were 109 compared to 3 and 117 compared to 2 in the tumors compared to normal tissue (Fig. 1A and 1B), and the expression of CXCR4 correlated with tumor grade (p < 0.001, ANOVA). Western blot of CXCR4 expression for a subset of primary tumors and normal cartilage showed similar results. (Fig. 1C).

### Effect of hypoxia on endogenous CXCR4 expression in chondrosarcoma cell line

**Figure 2:**
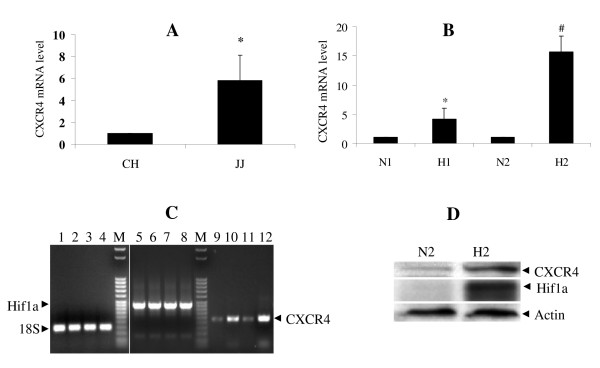
**CXCR4 expression during normoxia and hypoxia in chondrosarcoma cells**. A, CXCR4 mRNA expression in chondrosarcoma cell line JJ and normal chondrocytes (CH) in normoxia was analyzed by qRT-PCR. *, p < 0.02. B, CXCR4 mRNA expression in JJ cells in normoxia (N) and hypoxia (H) for 24 h (N1, H1) or 48 h (N2, H2) was quantitated by qRT-PCR. *, p < 0.05, compared to N1. #, p < 0.001, compared to N2. C, Representative electrophoresis of qRT-PCR products from B. Odd numbered lanes normoxia, even numbered lanes hypoxia; 24 and 48 h. Lanes 1-4: internal control (18S), lanes 5-8: Hif-1a, lanes 9-12: CXCR4. M is 50 bp molecular marker. D, Representative Western Blot of three independent experiments to detect CXCR4 and Hif-1a in JJ cells. Cell lysates were obtained after 48 h of culture in normoxia or hypoxia. Actin was used as loading control.

In chondrosarcoma cell line, the endogenous CXCR4 mRNA level was increased 6 fold compared to chondrocytes (Fig. 2A). Since tumors become hypoxic as they grow, and hypoxia increases expression of genes related to the malignant phenotype, we evaluated the expression of CXCR4 under hypoxic conditions. CXCR4 mRNA expression in JJ cells showed a progressive increase during hypoxia (2% O_2_) that reached 16 fold after 48 h (Fig. 2B, C). Western blot confirmed the qRT-PCR results (Fig. 2D).

### HIF-1a regulates CXCR4 expression

**Figure 3:**
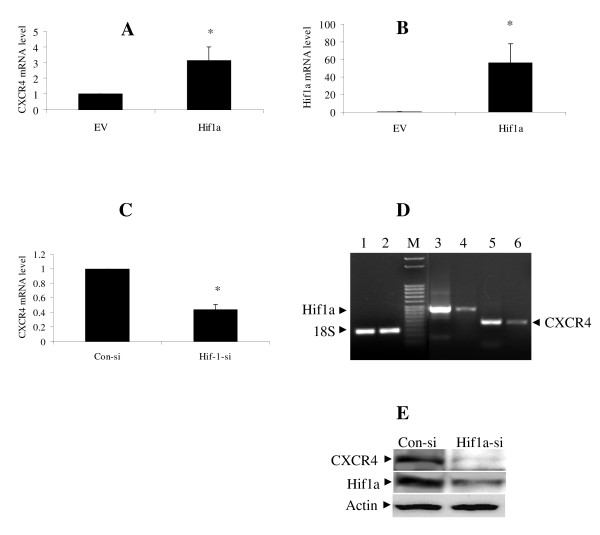
**Hif-1a regulates CXCR4 expression**. A and B, CXCR4 and HIF-1a expression after transfection with HIF-1a. JJ cells were transfected with HIF-1a expression construct or empty vector (EV). After 48 h, CXCR4 and HIF-1a expression was quantified by qRT-PCR; *, p < 0.02 and p < 0.01 respectively. C, D and E, CXCR4 and HIF-1a expression after HIF-1a knockdown. JJ cells were transfected with si-RNA directed against HIF-1a (HIF-1a si, lanes 2,4,6) or scramble siRNA (con-si, lanes 1,3,5) and CXCR4 and HIF-1a expression analyzed after 72 h of hypoxia with qRT-PCR (C, D) and Western blotting (E); *, p < 0.001. M is 50 bp molecular marker.

In order to assess if Hif-1a specifically mediates the increase in CXCR4 expression seen during hypoxia, HIF-1a transfection was performed. CXCR4 mRNA level increased by 3 fold relative to the empty vector control (Fig. 3A). Conversely, knockdown of Hif-1a with specific siRNA in JJ cultured in hypoxia decreased CXCR4 mRNA by 56% (Fig. 3C, D) and had the expected effect on Hif-1a expression (Fig. 3B, D). Western Blot showed the expressions of CXCR4 and Hif1a were reduced after Hif-1a knockdown during hypoxia. (Fig. 3E).

### Effect of hypoxia, HIF-1a and CXCR4 knockdown, and CXCR4 blockade on invasion

**Figure 4:**
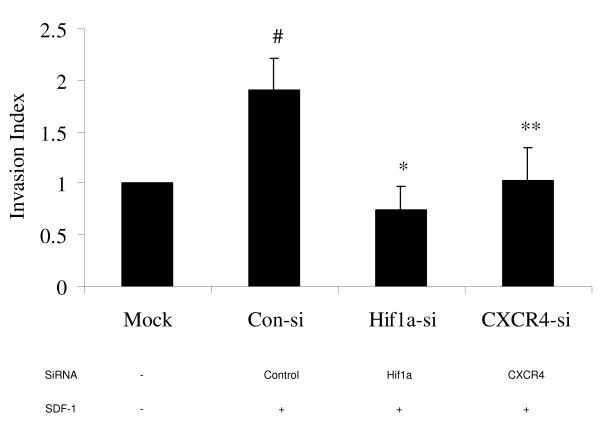
**Effect of Hif-1a and CXCR4 knockdown on the invasion of chondrosarcoma cells**. JJ cells were cultured in normoxia (mock) or hypoxia for 48 h after transfection with scramble siRNA (Con-si), or siRNA directed against Hif-1a or CXCR4. Invasion assay was then performed as described in Methods. #, p < 0.05, compared to Mock; *, p < 0.01, **, p < 0.05, compared with Con-si.

**Figure 5:**
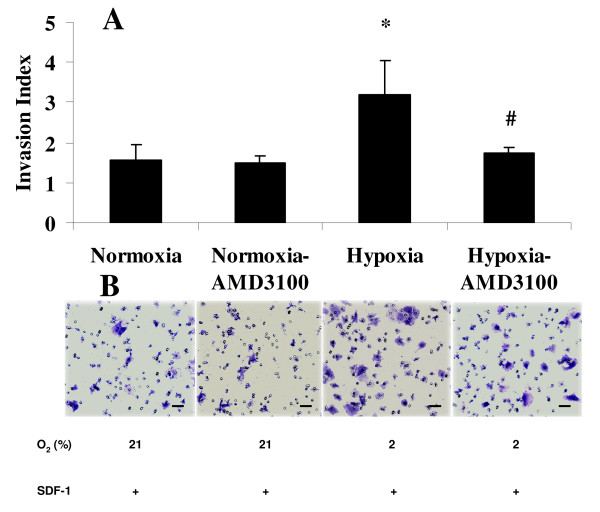
**Effect of hypoxia and CXCR4 inhibition on the invasion of chondrosarcoma cells**. JJ cells were cultured under normoxia or in hypoxia with 10 ng/ml SDF1 in the presence or absence of 0.1 μM of AMD3100 for 48 h (AMD3100 was added 2 h before SDF1) before performing the invasion assay, which was also performed during normoxia or hypoxia with or without AMD3100. *, p < 0.01; #, p < 0.05 compared to Hypoxia. Upper panel, bar graphs represent data from three independent experiments. Lower panel, representative photographs of stained cells on the lower side of matrigel membrane from one of three experiments. Original magnifications, 100×.

To test whether overexpression of CXCR4 drives chondrosarcoma cell metastasis, an *in vitro *cell invasion assay was performed. When cells were cultured in hypoxia and an SDF1 gradient, cell invasion increased 2 fold compared to normoxia, p < 0.05. Knockdown of Hif-1a or CXCR4 with specific siRNA completely blocked this increase in invasion that occurs during hypoxic culture (Fig. 4). Similarly, when the cells were pretreated with the CXCR4 inhibitor AMD3100, the hypoxia and SDF1 mediated increase in cell invasion was blocked, whereas AMD3100 had no effect during normoxia (Fig. 5).

### Hypoxia and CXCR4 signaling increase MMP1 expression and activity

**Figure 6:**
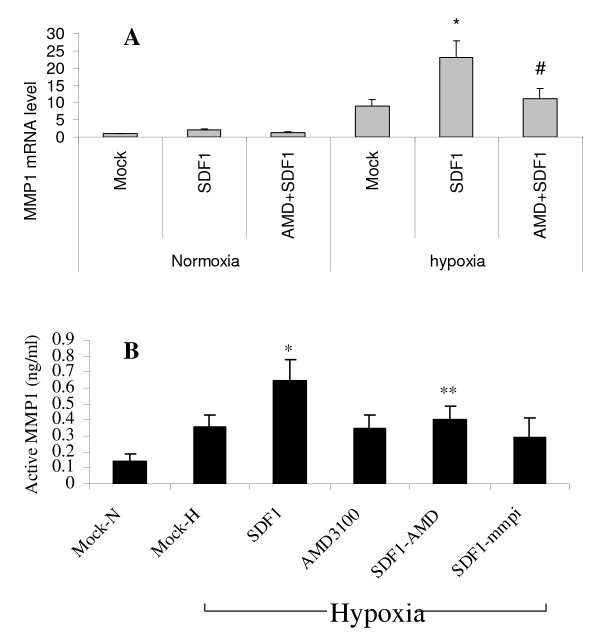
**SDF1 increases MMP1 mRNA level and MMP1 activity during hypoxia**. Tumor cells were cultured on matrigel with only medium (MOCK), SDF1 (10 ng/ml), AMD3100 0.1 μM, or SDF1+AMD3100 (AMD3100 was added 2 h before SDF1), or SDF1 + mmpi (MMPs inhibitor O-phenanthroline, 10 nM) for 24 h. Conditioned medium was then collected for ELISA and cells were harvested for RNA isolation. A, MMP-1 mRNA level was measured by qRT-PCR (*, p < 0.001, compared to Mock in hypoxia; #, p < 0.001, compared to SDF1 in hypoxia). B, active MMP-1 was measured in conditioned media by ELISA (*, p < 0.01, compared to Mock-H in hypoxia; **, p < 0.05, compared to SDF1 in hypoxia).

Cell invasion is in part mediated by matrix metalloproteinases. Figure 6 shows the effects of hypoxia and CXCR4 stimulation with SDF-1 or CXCR4 blockade with AMD3100 on MMP1 mRNA expression and secreted active MMP1 protein. Hypoxia increased MMP1 mRNA expression 9 fold which was further increased to 23 fold by SDF1 stimulation. There was no effect of SDF1 or AMD3100 during normoxia on MMP1 mRNA level. AMD3100 blocked the SDF1 mediated increase in MMP1 mRNA during hypoxia (Fig 6A). Similarly, hypoxia and SDF1 increased active MMP1 in conditioned media of cells cultured in hypoxia. AMD3100 had no effect during hypoxia without SDF1. AMD3100 in the presence of SDF1 had a similar effect as the MMP inhibitor O-phenanthroline (Fig 6B).

### Downstream effects of hypoxia and CXCR4/SDF-1 are mediated through ERK signaling

**Figure 7:**
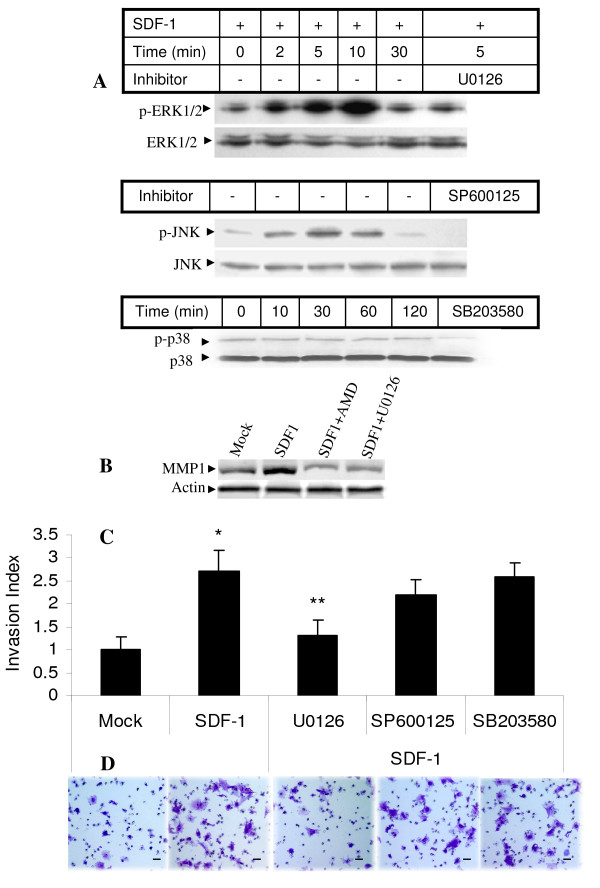
**MAP kinase is activated by SDF1 and ERK inhibitor reduces MMP1 production and CS cell invasion**. JJ cells were cultured with 10 ng/ml SDF1 or pretreated for 2 h with 10 μmol/L U0126, 40 nM SP600125, or 100 nM SB203580 in hypoxia. A, Whole cell lysates at indicated time points were subjected to Western blot analysis for total and phophorylated ERK, JNK, and p38. B, Cell lysates were used for Western blot analysis of total MMP-1 after 48 h, SDF1 10 ng/ml, AMD3100 0.1 μM, or U0126 10 μmol/L in hypoxia. C, JJ cells were cultured for 24 h in hypoxia with DMSO (Mock), SDF1 10 ng/ml alone or after 2 h pretreatment with MAP kinase inhibitors (U0126 10 μM, SP600125 40 nM, SB203580 2 μM), then invasion assay was performed for 72 h in hypoxia with SDF1 50 ng/ml in lower chamber only and inhibitors in both chambers. *, p < 0.01, compared to Mock; **, p < 0.05, compared to SDF-1. Bar graphs represent data from three independent experiments. D, representative photographs of stained cells on the lower side of matrigel membrane from one of three experiments in C. Original magnification 100×.

In order to assess the role of MAP kinases in CXCR4/SDF1 signaling, time course analysis of MAP kinase expression after SDF1 exposure was performed. SDF1 stimulation during hypoxia transiently increased phosphorylated ERK which reached a peak at 10 minutes. The increase in phosphorylated ERK could be inhibited by MEK (ERK) inhibitor U0126 (Fig. 7A). There was less effect of SDF1 on phosphorylated JNK and no effect on p38. SDF1 stimulation during hypoxia also increased MMP1 protein expression. Both the CXCR4 inhibitor AMD3100, the ERK inhibitor U0126, and ERK1/2 siRNA inhibited MMP1 protein expression (Fig. 7B, Additional file 1). The SDF1 mediated increase in cell invasion during hypoxia was also inhibited by U0126 and ERK1/2 siRNA, but not by the other MAP kinase inhibitors SP600125 and SB203580 (Fig 7C, D, Additional file 1).

## Discussion

A better understanding of the mechanisms underlying invasive behavior of a cancer is an important first step in developing improved treatment strategies. This study provides the first indication that CXCR4 is regulated by hypoxia and specifically HIF-1a in chondrosarcoma cells. We also show that increased CXCR4 signaling regulates expression of MMP1, a factor known to be involved with chondrosarcoma metastasis and a marker for poor prognosis[11]. Overexpression of CXCR4 has been reported in a variety of tumors, primarily carcinoma. In carcinoma, CXCR4 expression mediates metastasis to bone, which has relatively high levels of SDF1. In chondrosarcoma, it is possible that local SDF1 stimulates local tumor growth in a paracrine manner, and for those cells which gain access to the circulation, may also partially account for the tendency of these tumors to develop lung metastases, since the lung also contains high levels of SDF1. Factors such as MMP1 mediate local migration out of the microenvironment, ie stroma for carcinoma and bone for chondrosarcoma, and into the circulation. Factors such as CXCR4 mediate homing and growth at distant sites. Within sarcoma, CXCR4 expression has been detected in osteosarcoma [6,12]and recently in chondrosarcoma[13]. Our results confirm the expression of CXCR4 in both chondrosarcoma tissue and cell lines and also show that CXCR4 expression was higher in high grade tumors, that hypoxia and HIF-1a enhance CXCR4/SDF1 mediated invasion through upregulation of CXCR4 expression, and that CXCR4/SDF1 signaling increases invasion through ERK mediated increase in MMP1 expression and activity.

Hypoxia, primarily acting through HIF-1a, elicits a wide spectrum of changes in gene expression that contribute to the metastatic phenotype of cancer cells. Hypoxia and Hif-1a have been shown to upregulate CXCR4 in carcinomas such as lung cancer [14], oral squamous cell carcinoma[15], breast carcinoma [16], and renal cell carcinoma [17]. The mechanism of Hif-1a regulation of CXCR4 is through direct binding to the CXCR4 promoter [18]. Our results show that HIF-1a also upregulates CXCR4 in chondrosarcoma. Interestingly, during chondrogenic differentiation CXCR4 is downregulated[19]. Although chondrosarcoma share some markers of the cartilage phenotype, as cells become malignant, some repressed genes will be reexpressed. CXCR4 has been shown to be involved with cell migration and invasion in many systems. The data include *in vitro *invasion and migration assays as well as xenograft models of metastatic disease in which blockade of CXCR4 with drugs, peptides, or antibodies can inhibit development and growth of metastases. Independent of CXCR4, MMP1 has also been shown to be involved with tissue invasion and development of metastases. MMP1 is also known to be upregulated by hypoxia and HIF-1a in breast and lung cancer cells[20,21], and also by CXCR4 in Nk cells [22] and prostate cancer cells [23]. However, this project is the first to link the combined effects of HIF-1a on CXCR4 and MMP1 expression and the indirect effect of HIF-1a on MMP1 expression acting through CXCR4, which independently increases MMP1 in chondrosarcoma cells.

The role of MMP1 in chondrosarcoma invasion and its role as a poor prognostic indicator have been known for some time [11,24]. Inhibition of MMP1 with siRNA has been shown to decrease chondrosarcoma cell invasion [25-27]. We have shown that one mechanism of increased MMP1 in chondrosarcoma is mediated through CXCR4 signaling, which is amplified by hypoxia, and is mediated by ERK, but not other MAP kinases. siRNA directed against HIF-1a, CXCR4, ERK; CXCR4 blockade with AMD3100; or ERK inhibitor U0126 all efficiently inhibited the increase in invasion of chondrosarcoma cells during hypoxia. A previous study of CXCR4 in chondrosarcoma invasion during normoxia showed that CXCR signaling increased expression of alphavbeta3 integrin, also through ERK, and that alphavbeta3 integrin antibodies could also inhibit chondrosarcoma invasion in vitro[13]. Therefore, CXCR4 affects chondrosarcoma invasion through upregulation of multiple genes including alphavbeta3 integrin and MMP1. In other tumors and chondrosarcoma (Additional file 2), CXCR4 signaling upregulates other MMPs such as MMP-2, 8 and -9 and -13[28,29]. Since CXCR signaling upregulates multiple genes related to metastasis and since clinical MMP inhibition is not currently feasible, whereas CXCR4 blockade is possible with drugs such as AMD3100, CXCR4 may be a fruitful therapeutic target to inhibit some of the metastatic potential of chondrosarcoma cells.

## Conclusions

We present data that shows hypoxia mediated increase in MMP1 expression and chondrosarcoma invasion is partially mediated by CXCR4 signaling. CXCR4 blockade can inhibit the effects of hypoxia on MMP1 expression and chondrosarcoma invasion in vitro, suggesting that CXCR4 blockade could be a therapeutic target to inhibit chondrosarcoma invasion and metastasis. The effectiveness of this strategy requires in vivo confirmation.

## Methods

### Tissue

Articular cartilage, chondrosarcoma tissue, and cancellous bone were obtained from surgical specimens, and either preserved in RNAlater Solution (Applied Biosystems, Foster City, CA) or snap frozen in liquid nitrogen for later use. There were 8 articular cartilage specimens and 16 chondrosarcoma (3 grade I, 5 grade II, and 8 grade III). IRB approval was obtained.

### Cell lines and cell culture

Human chondrocytes isolated from normal adult articular cartilage and chondrosarcoma (CS) cell line JJ (a gift from Dr. Joel Block, Rush Medical School, Chicago, IL) were cultured in complete medium (40% DMEM, 40% MEM, 20% F12) with 10% FBS. All cells were cultured in a humidified incubator (NuAire Inc, Plymouth MN) under 5% CO_2 _and either normoxia (ambient oxygen) or hypoxia (2% O_2_) [30]. JJ was derived from a human grade II chondrosarcoma[31]. The drugs and inhibitors used were: AMD3100 (Sigma Chemical Company St. Louis, MO), human recombinant SDF-1 (R&D Systems, Minneapolis, MN), MMP inhibitor O-phenanthroline (Sigma), MAP kinase inhibitors: MEK1/2 inhibitor U0126 (Cell Signaling, Beverly, MA), JNK inhibitor SP600125 (A.G. Scientific Inc., San Diego, CA), p38 inhibitor SB203580 (BioMol International, Plymouth Meeting, PA) or DMSO (Sigma), solvent for the inhibitors.

### Transfections

Cells were transiently transfected with an expression construct for human Hif-1a in pcDNA3.1(+) vector (Invitrogen, Carlsbad, CA), (a gift from Hirouki Kato PhD, Tokyo Metropolitan Institute of Medical Science, Tokyo, Japan), or empty vector using Fugene HD (Roche, Indianapolis, IN) in 6 or 12 well plates 24 h after seeding. Cells were then incubated for 48 h and harvested for the following experiments.

### RNA interference (siRNA)

Cells were transfected with Hif-1a siRNA (Qiagen, Valencia, CA), CXCR4 siRNA (Dharmacon, Lafayette, CO), ERK1/2 siRNA(Cell Signaling Technology, Inc., Beverly, MA) or control siRNA by HiPerFect transfection reagent (Qiagen). RNA and protein were obtained 72 h after transfection for qRT-PCR and Western Blot analysis.

### Real-time RT-PCR (qPCR)

RNA was isolated from cells with RNAqueous Kit (Ambion, Austin, TX) or tissues with Trizol Reagent (Invitrogen). After treatment with TURBO DNase (Ambion), one microgram of RNA was reverse transcribed with random hexamers to obtain first-strand cDNA using iScript cDNA kit (Bio-Rad, Philadelphia, PA). The quantification of mRNA for Hif-1a, CXCR4, SDF-1, and MMP1 was performed by two-step real-time quantitative RT-PCR (Qiagen). Primers (5'-3') for Hif-1a were: forward, ctc aaa gtc gga cag cct ca; reverse, ccc tgc agt agg ttt ctg ct; for CXCR4, forward: gtc cac gcc acc aac ag, reverse: ctg ttg gtg gcg tgg ac; for SDF-1, forward: cgt gct ggt cct cgt gct gac; reverse: gct ttc tcc agg tac tcc tg; for MMP1, forward: gag caa aca cat ctg acc tac agg a; and reverse, ttg tcc cga tga tct ccc ctg aca; 18S was used as an internal control since it has been shown to be the optimal reference gene[32]. Amplification conditions were as follows: 2 min preincubation at 50°C, 10 minutes at 95°C for enzyme activation, and 40 cycles at 95°C denaturation for 10 s, 55°C annealing for 30 s (59°C for MMP1) and 72°C extension for 30 s. The comparative threshold cycle (Ct) method, i.e., 2^-ΔΔCt ^method was used for the calculation of fold amplification[33]. Each experiment was evaluated with three PCR reactions and each experiment was repeated three times. Data are presented as mean value ± SD.

### Western Blot Analysis

Protein from cell lysates of tissues or cells were separated via SDS-PAGE and probed with antibodies for CXCR4, MMP1, actin (Santa Cruz Biotechnology, Santa Cruz, CA); and antibodies for Hif-1a, p-ERK, ERK, p-JNK, JNK, p-p38, and p38 (Cell Signaling Technology, Inc.). Western Blot analyses were performed as previously described [10]. Protein concentrations were determined using the Bio-Rad Quick Start Bradford protein assay (Bio-Rad, Hercules, CA) and the equivalent of forty μg of protein were subjected to SDS-PAGE (Lonza Inc., Allendale, NJ).

### ELISA Assay

After treatment, cells were cultured O/N in FBS free medium and the conditioned media (CM) from CS cells was concentrated using Centricon-30 centrifugal filter device (Millipore, Billerica, MA). The amount of active MMP1 was detected using Human Active MMP1 Fluorescent Assay kit (R&D Systems, Minneapolis, MN) according to the manufacturer's instructions. Active MMP1 in the CM was measured in duplicate for each sample and normalized to the cell number at the end of the culture period. Each experiment was repeated three times.

### Tumor cell invasion assay

Invasive activity of CS cells was analyzed with matrigel coated BD Falcon Cell Culture Inserts (BD Biosciences, Bedford, MA). Briefly, 180 μl of BD Matrigel Matrix Growth Factor Reduced (BD Biosciences, Bedford, MA) diluted 1:3 with serum-free medium was used to coat 8 μm pore size 12-well inserts and incubated at 37°C for 2 h. After various treatments, during which cells are cultured O/N without FBS, the cells were harvested by trypsinization, counted, and resuspended in complete medium containing 1% FBS at a concentration of 10^6^/ml. 800 μl containing 8 × 10^5 ^cells were added to each of the upper wells. 1.5 ml of 5% FBS complete medium containing recombinant SDF1 (50 ng/ml, R&D Systems) was added to the lower wells. After incubating for 72 h in hypoxia, cells that had invaded across the membrane were stained with Cell Stain Solution (Millipore), washed, photographed, then lysed and cell number quantitated by absorbance at 560 nm on a standard microplate reader. The invasion index was calculated by normalizing to the number of cells invading when the lower well has no SDF1 or FBS.

### Statistics

All the experiments were repeated at least 3 times. Statistical analysis was performed with GraphPad Prism, v 3.0 (GraphPad Software, San Diego, CA). ELISA results and CXCR4 expression in different grades of chondrosarcoma were analyzed with one-way ANOVA. Post test comparisons were made with Bonferroni correction. Experiments with two groups were analyzed with the Student's t-test. The null hypothesis of no difference was rejected at a significance level of 5%.

## Competing interests

The authors declare that they have no competing interests.

## Authors' contributions

XS participated in the formation of the hypotheses, carried out the molecular biology experiments, and participated in interpretation of the data.

RMT participated in the formation of the hypotheses, in interpretation of the data, and drafted the manuscript.

QC participated in the formation of the hypotheses and in interpretation of the data.

LW participated in the formation of the hypotheses and in interpretation of the data.

All authors read and approved the final manuscript.

## Supplementary Material

Additional file 1 **Effect of ERK knockdown on MMP1 expression and invasion in chondrosarcoma cells**. A, Whole cell lysates from JJ cells were used for Western blot analysis of total MMP-1 after 48 h in hypoxia after ERK siRNA transfection. B, JJ cells were transfected with ERK siRNA. After 48 hours in hypoxia, invasion assay was performed as described in Methods. Click here for file

Additional file 2 **MMPs expressed in chondrosarcoma cells**. JJ cells were cultured in hypoxia 48 h without (mock) or with SDF-1 for 2 days. *, p < 0.02, **, p < 0.03, #, p < 0.04. Click here for file
