# Supplementary figures and images for: CXCR4/SDF1 mediate hypoxia induced chondrosarcoma cell invasion through ERK signaling and increased MMP1 expression
Source: Mol Cancer. 2010 Jan 26;9:17. doi: 10.1186/1476-4598-9-17 (PMC2825244; doi:10.1186/1476-4598-9-17)

## Slide 1
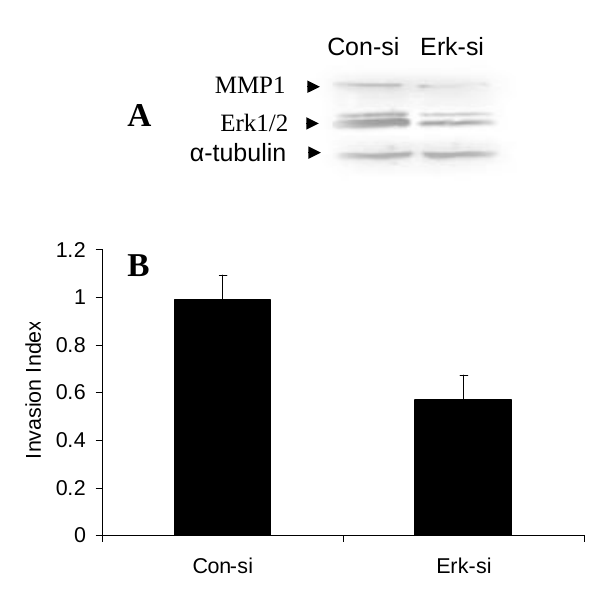

Con-si Erk-si
MMP1
Erk1/2
α-tubulin
A
B

Supplement: Additional file 1 — Effect of ERK knockdown on MMP1 expression and invasion in chondrosarcoma cells. A, Whole cell lysates from JJ cells were used for Western blot analysis of total MMP-1 after 48 h in hypoxia after ERK siRNA transfection. B, JJ cells were transfected with ERK siRNA. After 48 hours in hypoxia, invasion assay was performed as described in Methods. [file 1476-4598-9-17-S1.PPT]

## Slide 1
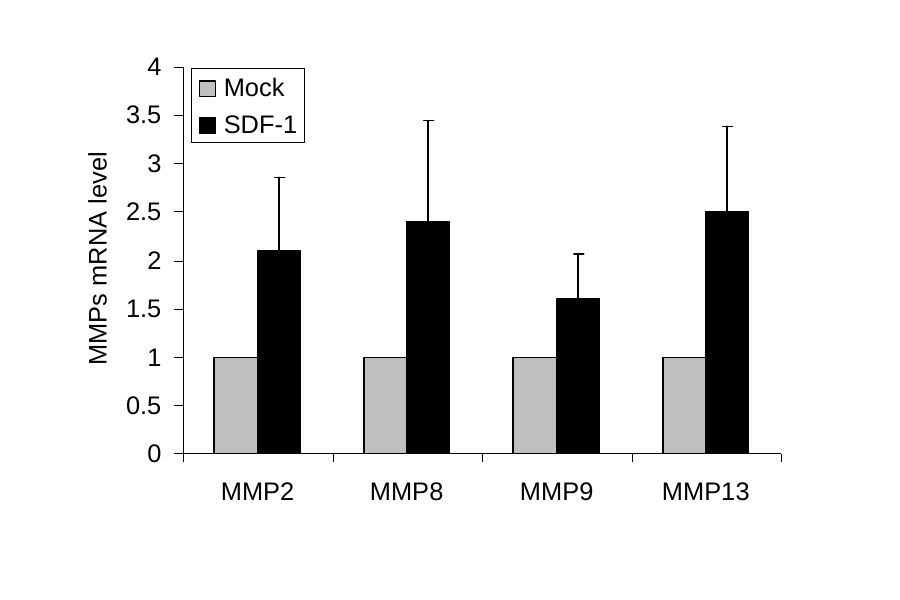

Supplement: Additional file 2 — MMPs expressed in chondrosarcoma cells. JJ cells were cultured in hypoxia 48 h without (mock) or with SDF-1 for 2 days. *, p < 0.02, **, p < 0.03, #, p < 0.04. [file 1476-4598-9-17-S2.PPT]
